# Oncolytic Viruses

**DOI:** 10.1155/2012/320206

**Published:** 2012-06-06

**Authors:** Nanhai G. Chen, Aladar A. Szalay, R. Mark L. Buller, Ulrich M. Lauer

**Affiliations:** ^1^Genelux Corporation, San Diego Science Center, San Diego, CA 92019, USA; ^2^Department of Radiation Oncology, Rebecca and John Moores Comprehensive Cancer Center, University of California, San Diego, CA 92093, USA; ^3^Department of Biochemistry, University of Wuerzburg, 97074 Wuerzburg, Germany; ^4^Rudolf Virchow Center for Experimental Biomedicine, University of Wuerzburg, 97078 Wuerzburg, Germany; ^5^Institute for Molecular Infection Biology, University of Wuerzburg, 97078 Wuerzburg, Germany; ^6^Department of Molecular Microbiology and Immunology, Saint Louis University School of Medicine, St. Louis, MO 63104, USA; ^7^Department of Gastroenterology and Hepatology, Medical University Hospital, 72076 Tübingen, Germany

Oncolytic viruses, by definition, are viruses that are capable of infecting and selectively replicating in cancer cells, eventually leading to cell death without harming healthy cells. The concept of using viruses to treat cancer dates back over a century. There was renewed interest in oncolytic virotherapy in the 1990s after the first genetically engineered oncolytic virus, a herpes simplex virus-1 thymidine kinase mutant, was reported in 1991. Over the last two decades great progress has been made in this field, and several oncolytic viruses have entered into clinical trials. This special issue on oncolytic viruses addresses some of the challenges that need to be overcome in order to achieve success in the clinic.

Oncolytic viruses can be delivered into cancer patients or preclinical animal models by a number of routes including intratumoral, intravenous, intrapleural, intraperitoneal delivery and hepatic artery infusion. Although intratumoral injection has been widely studied both preclinically and clinically, systemic delivery is the preferred method for treatment of the patient populations in greatest need of new efficacious therapies those with metastatic or inaccessible disease. The paper by M. S. Ferguson et al. summarizes the data from clinical trials using systemically delivered oncolytic viruses and then identifies barriers to this delivery approach and proposes solutions. 

The host immune response is a double-edged sword to the success of oncolytic virotherapy. The paper by C. A. Alvarez-Breckenridge et al. discusses the multifaceted relationship between oncolytic viruses and natural killer (NK) cells, a key component of innate immunity. The NK response to oncolytic virotherapy results in premature viral clearance while also mediating downstream antitumor immunity. Thus, it is critical to find an optimal approach to finely balance host antiviral and antitumoral immune responses.

The paper by M. H. Verheije and P. J. M. Rottier reviews the transduction targeting strategies currently employed to generate new oncolytic viruses with improved tumor specificity. The authors discuss advantages and limitations of each strategy by examples, and special attention is given to viruses expressing a bispecific adaptor protein consisting of two domains, one binding to the virion, the other to a cell surface protein of interest.

Hematopoietic stem cell transplant (HCT) is used to treat hematological malignancies; however, the contamination of healthy hematopoietic stem and progenitor cells with cancer cells presents a major challenge. The paper by S. Bais et al. provides a detailed review of using oncolytic viruses to treat hematological malignancies, and in particular, the use of oncolytic viruses to purge contaminating cancer cells for HCT. 

Many new oncolytic viruses have been generated through genetic engineering based on rational design. The paper by M. Bauzon and T. Hermiston presents an alternative method, that is, directed evolution that involves passaging diverse viruses under conditions that mimic the human cancer microenvironment. Using ColoAd1, the first directed evolution-derived oncolytic virus as an example, the authors underline the benefits of directed evolution and propose methods to “arm” these novel viruses and control their replication.

This special issue highlights some important recent findings in the field of oncolytic virotherapy and addresses challenges that the field is currently facing. Several oncolytic viruses have already entered, or will be entering randomized phase III trials. Amgen, the largest independent biotechnology company, has recently joined the field through acquisition of Biovex, highlighting the promising future of this therapeutic approach.

*Nanhai G. Chen* *Aladar A. Szalay* *R. Mark L. Buller* *Ulrich M. Lauer*

